# Glycemic Variability and Fluctuations in Cognitive Status in Adults With Type 1 Diabetes (GluCog): Observational Study Using Ecological Momentary Assessment of Cognition

**DOI:** 10.2196/39750

**Published:** 2023-01-05

**Authors:** Luciana Mascarenhas Fonseca, Roger W Strong, Shifali Singh, Jane D Bulger, Michael Cleveland, Elizabeth Grinspoon, Kamille Janess, Lanee Jung, Kellee Miller, Eliza Passell, Kerry Ressler, Martin John Sliwinski, Alandra Verdejo, Ruth S Weinstock, Laura Germine, Naomi S Chaytor

**Affiliations:** 1 Department of Community and Behavioral Health Elson S Floyd College of Medicine Washington State University Spokane, WA United States; 2 Old Age Research Group (PROTER), Department and Institute of Psychiatry, University of Sao Paulo Sao Paulo Brazil; 3 Institute for Technology in Psychiatry, McLean Hospital Belmont, MA United States; 4 Department of Psychiatry, Harvard Medical School Boston, MA United States; 5 Department of Medicine, State University of New York Upstate Medical University Syracuse, NY United States; 6 Department of Human Development, Washington State University Pullman, WA United States; 7 Jaeb Center for Health Research Tampa, FL United States; 8 The Silvio O Conte Center for Stress Peptide Advanced Research, Education, & Dissemination Center (SPARED), Department of Psychiatry, McLean Hospital, Harvard Medical School Boston, MA United States; 9 Department of Human Development and Family Studies, The Pennsylvania State University State College, PA United States; 10 Center for Healthy Aging, Pennsylvania State University State College, PA United States

**Keywords:** ecological momentary assessment, type 1 diabetes, cognitive variability, digital neuropsychology, digital technology, remote assessment, continuous glucose monitoring, cognition, diabetes, physiological, behavioral, psychological, cognitive, adults, glucose, data, study design, assessment, sample, hypoglycemia

## Abstract

**Background:**

Individuals with type 1 diabetes represent a population with important vulnerabilities to dynamic physiological, behavioral, and psychological interactions, as well as cognitive processes. Ecological momentary assessment (EMA), a methodological approach used to study intraindividual variation over time, has only recently been used to deliver cognitive assessments in daily life, and many methodological questions remain. The Glycemic Variability and Fluctuations in Cognitive Status in Adults with Type 1 Diabetes (GluCog) study uses EMA to deliver cognitive and self-report measures while simultaneously collecting passive interstitial glucose in adults with type 1 diabetes.

**Objective:**

We aimed to report the results of an EMA optimization pilot and how these data were used to refine the study design of the GluCog study. An optimization pilot was designed to determine whether low-frequency EMA (3 EMAs per day) over more days or high-frequency EMA (6 EMAs per day) for fewer days would result in a better EMA completion rate and capture more hypoglycemia episodes. The secondary aim was to reduce the number of cognitive EMA tasks from 6 to 3.

**Methods:**

Baseline cognitive tasks and psychological questionnaires were completed by all the participants (N=20), followed by EMA delivery of brief cognitive and self-report measures for 15 days while wearing a blinded continuous glucose monitor. These data were coded for the presence of hypoglycemia (<70 mg/dL) within 60 minutes of each EMA. The participants were randomized into group A (n=10 for group A and B; starting with 3 EMAs per day for 10 days and then switching to 6 EMAs per day for an additional 5 days) or group B (N=10; starting with 6 EMAs per day for 5 days and then switching to 3 EMAs per day for an additional 10 days).

**Results:**

A paired samples 2-tailed *t* test found no significant difference in the completion rate between the 2 schedules (t_17_=1.16; *P*=.26; Cohen *d_z_*=0.27), with both schedules producing >80% EMA completion. However, more hypoglycemia episodes were captured during the schedule with the 3 EMAs per day than during the schedule with 6 EMAs per day.

**Conclusions:**

The results from this EMA optimization pilot guided key design decisions regarding the EMA frequency and study duration for the main GluCog study. The present report responds to the urgent need for systematic and detailed information on EMA study designs, particularly those using cognitive assessments coupled with physiological measures. Given the complexity of EMA studies, choosing the right instruments and assessment schedules is an important aspect of study design and subsequent data interpretation.

## Introduction

### Ecological Momentary Assessment

Ecological momentary assessment (EMA) is a methodological approach used to study intraindividual variation over time, using repeated assessments of behavioral, physiological, and psychological processes during regular daily activities using electronic devices [1]. In recent years, the increasing reach of technology, have dramatically increased the feasibility and sophistication of approaches that use EMA [2,3]. The use of EMA overcomes many limitations of traditional study designs, such as retrospective response bias and undetected environmental influences on behavior, as well as allowing for real-time intervention [4,5]. However, as a relatively recent methodology, there are some challenges to the use of EMA, including the relative scarcity of specific evidence-based methodological guidelines for conducting studies. Moreover, few studies have described EMA methodology in sufficient detail for replication [6]. This lack of methodological guidance is particularly notable in the assessment of cognitive performance via EMA.

### EMA for Individuals With Type 1 Diabetes

Individuals with type 1 diabetes (T1D) are a particularly important target population for EMA study designs given their vulnerabilities to and interactions among multiple dynamic physiological, behavioral, and psychological processes. T1D is a chronic autoimmune disease characterized by the destruction of insulin-producing β-cells in the pancreas, causing hyperglycemia [7] and requiring the use of exogenous insulin. Variations in blood glucose, which occur over the course of minutes to hours, are associated with short-term cognitive variability [8-12] in controlled studies and may indirectly impact psychological and other physiological states [13-15]. We are aware of 3 other ongoing studies that are using continuous glucose monitoring (CGM) coupled with EMA in adults with T1D: The Function and Emotion in Everyday Life with T1D study [16], Hypoglycaemia – Redefining Solutions for better lives project [17], and Towards a Better Understanding of Diabetes Distress, Depression and Poor Glycaemic Control study [18]. One of these studies does not include ambulatory cognitive assessment [18], and the other 2 have not yet reported results [16,17].

The primary goal of the Glycemic Variability and Fluctuations in Cognitive Status in Adults with Type 1 Diabetes (GluCog) study is to characterize the relationship between glycemic excursions and cognitive functioning in adults with T1D, with the secondary goal of determining how psychological state and diabetes-related factors mediate and moderate this relationship. The study, led by principal investigators Dr Laura Germine of McLean Hospital and Dr Naomi Chaytor of Washington State University, uses the EMA of cognitive performance and self-report data, coupled with blinded CGM. Four endocrinology centers (SUNY Upstate Medical University, University of Pennsylvania, Mayo Clinic, and AdventHealth Diabetes Institute), with central clinical site coordination by the Jaeb Center for Health Research, are currently recruiting participants for this study.

### Aims of the Study

Here, we report the results of an initial EMA optimization pilot study of 20 participants with T1D. This optimization pilot was conducted before the finalization of the GluCog protocol to determine the appropriate EMA frequency for the detection of hypoglycemia and EMA completion rate and to refine our cognitive EMA battery. We describe how the initial optimization pilot results guided the design of the main GluCog study, which was launched in September 2020 and is ongoing. Although this study collected CGM data in adults with T1D, similar methodological considerations are applicable to any semicontinuous physiological or behavioral data collection coupled with discrete EMA data (eg, actigraphy, heart rate, and continuous electroencephalogram monitoring). To establish the optimal EMA frequency, we evaluated 2 EMA schedules (6 EMAs per day for 5 days vs 3 EMAs per day for 10 days) to determine which schedule (1) captured the highest number of hypoglycemic episodes within 60 minutes before each EMA and (2) resulted in higher EMA completion rates. We focused on EMA after hypoglycemia (rather than hyperglycemia) because of the established association with cognitive performance in controlled studies and given that hypoglycemia is less frequent than hyperglycemia [19,20].

## Methods

### Participants

In total, 20 adults with T1D were enrolled in the optimization study between February 2020 and May 2020 from SUNY Upstate Medical University based on the inclusion criteria that they must: be ≥25 years of age, be diagnosed with T1D, have T1D for >1 year, be fluent in English, have understood the EMA protocol and agreed to comply with it to the best of their ability, and have 24-hour access to a personal smartphone with reliable internet access. Exclusion criteria included the following: inability to complete cognitive assessments owing to significant visual, motor, hearing, or cognitive impairment; any medical or psychiatric condition or treatment that was determined by the principal investigators to interfere with the completion of the study; current use of real-time CGM; inability to complete EMA assessments during the study period (eg, night shift work, planned travel across time zones, or occupation that does not reliably allow time to complete assessments within a reasonable period).

### Materials

#### Baseline Assessment

Baseline cognitive tasks and psychological questionnaires were completed by all the participants via their smartphones, tablets, or computers through a secure website (TestMyBrain.org [21]; TMB) managed by the study staff at McLean Hospital. The baseline assessment duration was approximately 60 minutes. Tasks were selected based on the recommendations from the Core Neuropsychological Measures for Diabetes and Obesity Trials [22]. Full-length versions of all cognitive EMA (see below) were also included. For a complete list of the baseline assessments and constructs measured, see Textbox 1.

**Baseline assessments and constructs measured.**

**Baseline questionnaires, approximately 40 minutes**

- General questionnaire
  Demographic characteristics, employment, sleep and wake times in a typical week, and work time
- Mental Health Questionnaire
  Questionnaire assessing cross-cutting symptoms for psychopathology based on the Diagnostic and Statistical Manual of Mental Disorders, Fifth Edition [23,24]. This is a 6-item questionnaire assessing possible broad psychopathology. It takes approximately 2 minutes.
- Pittsburgh Sleep Quality Index [25]
  Questionnaire assessing sleep duration and quality over a 1-month interval. It takes approximately 5 minutes.
- Snoring, tiredness, observed apnea, high BP, BMI, age, neck circumference, and male gender (STOP-Bang) Questionnaire [26]
  Questionnaire assessing obstructive sleep apnea risk consisting of 8 questions that take approximately 2 minutes. STOP-BANG sensitivity is of 93% and 100% for detecting moderate and severe sleep apnea [27].
- Functional Activities Questionnaire [28]
  Questionnaire assessing instrumental activities of daily living consisting of 10 items and administered to an informant
- Patient Health Questionnaire-8 (PHQ-8) [29]
  An 8-item questionnaire assessing depression symptoms that takes approximately 5 minutes
- Generalized Anxiety Disorder (GAD-7) [30]
  A 7-item self-report scale assessing generalized anxiety symptoms that takes approximately 5 minutes
- Global perceived stress scale [31]
  Questionnaire assessing the chronic experiences of stress. It is a 10-item scale measuring the degree to which situations are appraised as stressful. It takes approximately 5 minutes.
- World Health Organization Alcohol, Smoking, and Substance Involvement Screening Test (ASSIST) [32]
  Screening for alcohol consumption, smoking, and other substance use throughout lifetime and during the latest 3 months at the time of assessment. It takes approximately 3 minutes.
- Quality of Life in Neurological Disorders (Neuro-QoL)—Cognitive Function Short Form [33]
  An 8-item questionnaire assessing self-reported cognitive problems in daily life. Neuro-QoL provides a common metric for use across patient groups in different studies [34]. It takes approximately 5 minutes.
- Coronavirus Health Impact Survey (CRISIS) [35]
  Questionnaire covering key domains relative to mental distress and resilience, which assess self-reported impact owing to the COVID-19 pandemic. It was demonstrated to have good feasibility, reliability, and construct validity in large pilot samples in the United States and United Kingdom [35]. It takes approximately 10 minutes.

**Baseline cognitive assessment, approximately 30 minutes**

- TestMyBrain.org (TMB) Matrix Reasoning
  Cognitive test assessing general cognitive ability and nonverbal reasoning. Participants solve a series of visual puzzles.
- TMB Vocabulary
  Cognitive test assessing verbal reasoning. Participants indicate which of 5 words is the closest in meaning to a target word.
- TMB Simple Reaction Time
  Cognitive test assessing basic psychomotor speed. Participants press a button every time a green square appears on screen.
- TMB Letter-Number Switching
  Cognitive test assessing cognitive flexibility and task switching. Participants indicate which response fits the instruction cue shown on screen.
- TMB Visual Paired Associates Memory
  Cognitive test assessing visual memory. Participants learn a set of picture pairs and have to indicate which pictures go together based on the set they learned.
- TMB Delay Discounting
  Cognitive test assessing decision-making. Participants indicate whether they would prefer differing amounts of hypothetical money now or in the future.

**Baseline full-length cognitive ecological momentary assessment tests, approximately 10 minutes**

- TMB Flicker Change Detection (Flicker)
  Cognitive test assessing visual working memory. Participants view a series of visual scenes with blue and yellow dots. One of the dots is changing color from blue to yellow. Participants are asked to indicate the dot that is changing color.
- TMB Multiple Object Tracking (MOT)
  Cognitive test assessing visuospatial working memory. Participants track dots as they move across the screen.
- TMB Paced Serial Addition Test (PSAT)
  Cognitive test assessing sustained attention. Participants indicate whether last 2 numbers add up to >10 or <10.
- TMB Gradual Onset Continuous Performance Test (GradCPT)
  Cognitive test assessing sustained attention. Participants see a series of city or mountain scenes and are asked to press a button whenever they see a city scene and withhold a response whenever they see a mountain scene.
- TMB Digit Symbol Matching (DSM)
  Cognitive test assessing psychomotor processing speed. Participants have to match a set of symbols to the numbers 1, 2, or 3 based on a key presented on screen.
- TMB Choice Reaction Time (Choice RT)
  Cognitive test assessing psychomotor processing speed

#### Cognitive EMA

Cognitive tasks selected for the optimization pilot were based on prior TestMyBrain [21] website-collected data showing good sensitivity and internal reliability for ultrabrief versions (reliability of 0.4 or higher for one 30-60-second testing occasion [36]) across alternate forms suitable for EMA, theoretical association with blood glucose excursions, and prior use in brain health research [37,38] (refer to Multimedia Appendix 1 for a complete list of the EMA questions and cognitive tasks [37-44]; Cognitive EMA selection in the *Results* section). Two tests of processing speed (Brief TMB Choice Reaction Time and Brief TMB Digit Symbol Matching [DSM]), cognitive control or sustained attention (Brief TMB Gradual Onset Continuous Performance Test [GradCPT] and Brief TMB Paced Serial Addition Test), and visual working memory (Brief TMB Multiple Object Tracking [MOT] and Brief TMB Flicker) were selected for evaluation during the optimization phase. To maintain a total EMA duration of <5 minutes, 3 tests (1 from each cognitive domain) were administered during each EMA and counterbalanced across EMAs to ensure equal exposure to all tasks across each EMA frequency period (ie, 3 and 6 EMAs per day). Participants who completed <50% of the total EMAs were excluded from the data analyses.

For all the tasks described in Multimedia Appendix 1, 21 alternate forms were generated based on validated algorithms to minimize practice effects. Versions differed in trial order or items to be remembered but not in any substantive characteristics (eg, task length, parameters, and stimuli). The cognitive EMA tasks were performed on a personal smartphone using a dedicated mobile TestMyBrain [21] study site.

#### Blinded CGM

A blinded Dexcom G6 Personal CGM System (Dexcom CGM). The CGM system (Food and Drug Administration–approved) was inserted and worn for a minimum of 10 days and a maximum of 20 days (a second sensor was sent home with the participant). The CGM system consisted of a sensor (plus an additional sent home for insertion after 10 days), transmitter, and receiver (set to blinded mode before assigning to the participant). Participants with <3 days of CGM data (72 h) were excluded from the data analyses.

#### Hypoglycemia Criteria

CGM data were coded for the presence of hypoglycemia within 60 minutes before the start of each EMA. This time frame was chosen based on insulin clamp studies demonstrating cognitive recovery within 40 to 90 minutes of return to euglycemia [45]. On the basis of recent consensus criteria recommendations [46], we operationalized *hypoglycemia* as >15 consecutive minutes with a sensor glucose value of <70 mg/dL. At least 2 sensor values <70 mg/dL that are ≥15 minutes apart, plus no intervening values of >70 mg/dL, are required to define a hypoglycemic event. The end of the hypoglycemic event is defined as a minimum of 15 consecutive minutes with a sensor glucose concentration of >70 mg/dL. At least 2 sensor values of >70 mg/dL that are ≥15 minutes apart, with no intervening values of <70 mg/dL, are required to define the end of an event. We chose the sensor glucose value of <70 mg/dL to maximize the likelihood of these events occurring within 60 minutes of EMA. Hypoglycemic events were excluded if there were missing values or discontinuous jumps between adjacent measures (indicating potential sensor error) [47].

#### Passive Measures

The metadata of the browser, screen size, and operating system were captured to assist in the interpretation of cognitive data, as data quality is critically dependent on the accurate capture of device characteristics that can confound smartphone–based cognitive assessments [48].

### Ethics Approval

The GluCog optimization pilot study was conducted in compliance with ethical principles that have their origin in the Declaration of Helsinki, including Regulations for the Protection of Human Participants of Research, and the standards of Good Clinical Practice. This study was approved by the Jaeb Center for Health Research Institutional Review Board. All the participants provided written informed consent.

### Procedure

#### Overview

**Figure 1:**
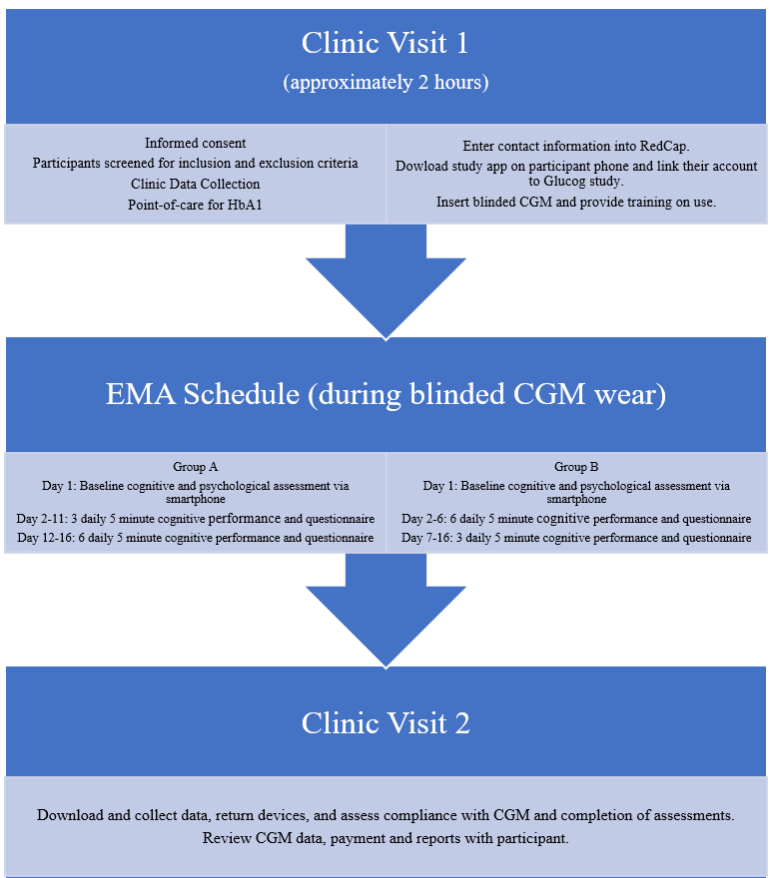
Schematic of study design. CGM: continuous glucose monitoring; GluCog: Glycemic Variability and Fluctuations in Cognitive Status in Adults with Type 1 Diabetes study; HbA_1c_: hemoglobin A_1c_.

For schematic of the overall study design, see Figure 1. Clinic data collection consisted of physical exam (height, weight, blood pressure, heart rate, and waist and neck circumference) conducted by a medical provider; demographic and socioeconomic information; medical record review and patient-reported diabetes history, including age at diagnosis, severe hypoglycemia history, and diabetic ketoacidosis history; current diabetes and other medications; insulin administration method; hypoglycemia awareness assessment scale; and other medical conditions. Hemoglobin A1c was newly collected if not done clinically within 3 months. Participants were reimbursed for completion of the following study components: clinic visit 1, baseline assessment, each EMA (up to a maximum of US $30), an extra bonus for completion of >80% EMA and clinic visit 2). To be included in data analysis, the participant must have completed ≥50% of EMAs.

#### EMA Schedule Randomization

To evaluate the impact of EMA administration schedules on completion rates, detection of hypoglycemia, and cognitive variability, we randomized participants (1:1) into one of two counterbalanced EMA frequency groups. Group A began with low-frequency or long-duration EMA (3 EMAs/day over 10 days), followed by high-frequency or short-duration EMA (6 EMAs/day over 5 days). Group B began with high-frequency or short-duration EMA (6 EMAs/day over 5 days), followed by low-frequency or long-duration EMA (3 EMAs/day over 10 days). All EMAs were delivered between 9 AM and 9 PM local time to minimize the effects of varying sleep schedules and sleep inertia on performance. Each of the 6 mobile tests was administered 30 times to each participant (15 times in the 6 EMA/day schedule and 15 times in the 3 EMA/day schedule), with each EMA occasion including one of two tests from each of the 3 cognitive domains.

#### EMA Schedule

**Figure 2:**
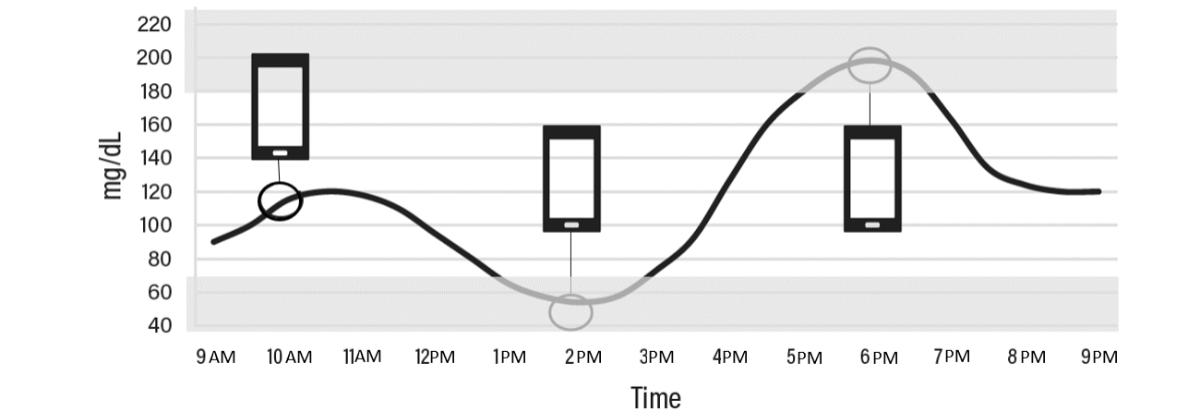
Cognitive ecological momentary assessment (EMA) coupled with continuous glucose monitoring (CGM)—for example, schedule with 3 EMAs per day. CGM data collected every 5 minutes. Smartphone icons represent hypothetical 3 times a day EMA assessment.

**Figure 3:**
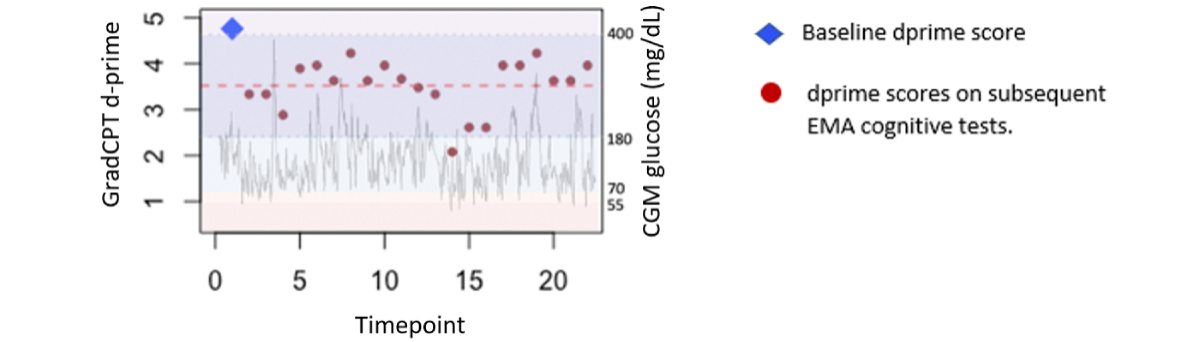
Example of ecological momentary assessment (EMA) coupled with continuous glucose monitoring (CGM) data for a single participant. GradCPT: Gradual Onset Continuous Performance Test.

The participants completed all the EMAs on their personal smartphones. On day 2 of the CGM sensor wear, following completion of the baseline assessment, participants were sent a smartphone notification to complete an onboarding EMA consisting of detailed instructions and practice trials for the cognitive EMA tasks. Figures 2 and 3 show a diagram of the 3 EMAs per day coupled with CGM. Each EMA consisted of patient-reported questions and brief cognitive tasks with a total duration of 5 minutes, occurring 3 to 6 times a day (refer to EMA Schedule Randomization) on days 3 to 18. The formal EMA schedule began in the morning of day 3 after wearing CGM sensor for 2 days, followed by multiple daily assessments for 15 days. Each participant was sent push notifications containing a link to the EMA battery. Notifications were sent at random within prespecified time windows (for the 3 EMA/day schedule, a notification was sent between 9:00 AM and 12:59 PM, 1 PM and 4:59 PM, and 5 PM and 9 PM; for the 6 EMA/day schedule, a notification was sent between 9 AM and 10:59 AM, 11 AM and 12:59 PM, 1 PM and 2:59 PM, 3 PM and 4:59 PM, 5 PM and 6:59 PM, and 7 PM and 9:00 PM). Participants had up to 30 minutes to start each EMA from the time the notification was delivered. Participants received a reminder text message when 25 minutes had elapsed, stating that it was the final chance to complete the EMA.

#### Technical Support

Technical support was provided during working business hours, that is, 9 AM to 5 PM Eastern Time from Monday to Friday. In addition to the general availability to answer questions, technical support staff monitored participant assessment completion and intervened during the initial days following study enrollment. On days 1 and 2 of the study, participants were expected to complete a baseline assessment (link sent through email) and onboarding assessment (sent through app notification). If the participants had not completed both assessments by 3 PM Eastern time on day 2, they were sent a text reminder to complete these assessments as soon as possible. In this reminder, participants were told to contact us via Google Voice SMS and laboratory phone number if they experienced any technical problems. Technical problems such as an error in a survey link, issues in loading the webpage, and session timeout errors would result in a clinical research assistant troubleshooting with the participant via Google SMS. If the issue could not be resolved over SMS text messaging, a phone call was made to the participant and the participant’s clinic coordinator and the issue was elevated to the team’s software engineer. In general, problems typically arose within the first 48 to 72 hours of study enrollment and could usually be resolved over SMS text messaging. In rare cases where the issue could not be resolved, such as smartphone capability issue, a participant could be unenrolled from the study. For the remainder of the study, on days 4, 8, and 12, participants received standard status updates regarding their completion rate and follow-up on previously reported issues.

### Test Selection for Main Study: Psychometric Analyses of Pilot Data

Individual cognitive tests were evaluated for inclusion in the main study based on three sequential criteria: (1) completion rates and usability (eg, minimal participant burden or technical barriers), (2) minimal restriction of range, and (3) good between-person reliability in mobile format.

To calculate the between-participant reliability of each mobile cognitive test, we separately calculated the test scores for even and odd trials. For each test, unconditional multilevel mixed models were used to predict performance on each half of each mobile test, with random effects of EMA numbers nested within participants. Fitting these models allowed partitioning of variance to between- and within-person effects, which we entered into the following equation [36]:

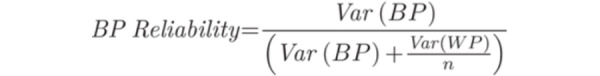

where Var (BP) is the total variance in scores between participants, Var (WP) is the variance in scores within participants (ie, variance between EMA sessions and residual variance), and n is the total number of measurements. For each test, we set n equal to the average number of measurements per participant, with a maximum possible n of 60 (a measurement of each half of the 30 mobile tests). CIs for the between-person reliability of each test were calculated using 10,000 bootstrap samples, with resampling at the participant level. Task psychometrics were compared across tasks and 3 EMA/day and 6 EMA/day schedules.

## Results

### Cognitive EMA Selection

Here, we present the results that led to the choice of cognitive EMA tests used during the optimization pilot phase. Table 1 is based on the previous data collected from the TestMyBrain.org [21] website showing good sensitivity and internal reliability (see Multimedia Appendix 2 for score distribution across tests). All tests exceeded our reliability threshold of 0.4 for a single 30-to-60-second testing occasion. For clarity, the EMA versions of the TMB tests are designated as *brief*.

**Table 1:** Initial reliability data for brief cognitive tests, based on the data collected from the digital research platform TestMyBrain.org [21].

Outcome	TestMyBrain.org sample		
	n	Mean (SD)	Reliability of the EMA^a^ length test^b^
Brief TMB^c^ Choice RT^d^—median RTc (ms)	12,939	961 (342)	0.93
Brief TMB DSM^e^—median Tc (ms)	7095	1005 (374)	0.81
Brief TMB Flicker—medianRTc (ms)	11,869	4873 (2461)	0.62
Brief TMB GradCPT^f^—d-prime	5039	2.92 (0.94)	0.82
Brief TMB MOT^g^—accuracy	10,703	69.4 (11.6)	0.67
Brief TMB PSAT^h^—accuracy	9900	72.8 (18.8)	0.75

^a^EMA: ecological momentary assessment.

^b^To calculate internal reliability, we fit unconditional multilevel mixed models predicting performance on each half of the test with a random effect of participants. The variance between and within participants was again entered into the same equation, with n=2 for the TestMyBrain.org sample (ie, the 2 halves of the single testing session). Note that for the TestMyBrain.org sample, this produces the same reliability value as the Spearman-Brown corrected split-half reliability of the even and odd trials.

^c^TMB: TestMyBrain.org.

^d^RT: Reaction Time.

^e^DSM: Digit Symbol Matching.

^f^GradCPT: Gradual Onset Continuous Performance Test.

^g^MOT: Multiple Object Tracking.

^h^PSAT: Paced Serial Addition Test.

### Optimization Pilot

The optimization pilot sample (N=20) was recruited from a single clinic site (SUNY Upstate Medical University) from February 2020 to April 2020. Initially, 30% (6/20) of participants were enrolled before the widespread COVID-19 lockdowns. We modified the protocol, obtained International Review Board approval for fully remote study visits, and enrolled the remaining optimization study sample participants remotely. Subsequently, 50% (10/20) of participants were randomized into EMA group A (starting with 3 EMAs per day for 10 days and then switching to 6 EMAs per day for an additional 5 days), and 50% (10/20) of participants were randomized to the EMA group B (starting with 6 EMAs per day for 5 days and then switched to 3 EMAs per day for an additional 10 days). However, of the 10 participants, 2 (20%) participants in group A were excluded from the analyses as they completed <50% of the EMAs (prespecified minimum EMA completion). One participant reported sleeping through all morning EMAs, and the other participant declined to troubleshoot technical issues.

Completion of the remaining sample (N=18) was good across both EMA frequency schedules (3 and 6 EMAs per day) and randomization groups—groups A and B (Table 2). The reported mean for each test was calculated by first computing the mean of each participant across all completed EMA tests and then calculating the average and SD of those participant-level means.

Between-participant reliability was greater for the GluCog optimization pilot sample than for the TestMyBrain.org [21] sample (Table 1), consistent with the repeated testing approach of the EMA design, increasing the measurement precision of between-participant differences relative to a single testing session.

The psychometric characteristics of the mobile cognitive tests were similar across both EMA schedules (Table 3).

**Table 2:** Means, SDs, and reliability of all tests across ecological momentary assessment schedules.

Test	GluCog^a^ optimization sample		
	N	Mean (SD)	Mean reliability (range)
Brief TMB^b^ Choice RT^c^—medianRTc (ms)	18	731 (114)	0.99 (0.93-0.99)
Brief TMB DSM^d^—medianRTc (ms)	18	839 (135)	0.99 (0.97-0.99)
Brief TMB Flicker—medianRTc (ms)	18	3768 (1771)	0.98 (0.90-0.99)
Brief TMB GradCPT^e^—d-prime	18	3.06 (0.36)	0.91 (0.81-0.94)
Brief TMB MOT^f^—accuracy	18	71.7 (7.1)	0.96 (0.92-0.98)
Brief TMB PSAT^g^—accuracy	18	83.4 (23.7)	0.99 (0.91-0.998)

^a^GluCog: The Glycemic Variability and Fluctuations in Cognitive Status in Adults with Type 1 Diabetes.

^b^TMB: TestMyBrain.org.

^c^RT: Reaction Time.

^d^DSM: Digit Symbol Matching.

^e^GradCPT: Gradual Onset Continuous Performance Test.

^f^MOT: Multiple Object Tracking.

^g^PSAT: Paced Serial Addition Test.

**Table 3:** Performance for each cognitive test, ecological momentary assessment (EMA) completion rate, and hypoglycemia episodes captured during the 3 EMA per day schedule and the 6 EMA per day schedule.

Outcome	3 EMAs per day			6 EMAs per day		
	N	Mean (SD)	Reliability	N	Mean (SD)	Reliability
Brief TMB^a^ Choice RT^b^—medianRTc (ms)	—^c^	728 (116)	0.98	—	733 (124)	0.99
Brief TMB DSM^d^—medianRTc (ms)	—	833 (146)	0.99	—	844 (142)	0.98
Brief TMB Flicker—medianRTc (ms)	—	3809 (1606)	0.95	—	3727 (2146)	0.98
Brief TMB GradCPT^e^—d-prime	—	3.04 (0.45)	0.90	—	3.08 (0.30)	0.67
Brief TMB MOT^f^—accuracy	—	72.5 (7.4)	0.94	—	71.0 (7.0)	0.92
Brief TMB PSAT^g^—accuracy	—	83.6 (24.0)	0.98	—	83.3 (23.8)	0.98
EMA completion rate	—	85.7 (10.6)	—	—	81.7 (14.9)	—
Total hypoglycemic events (whole sample)	102	5.7 (5.6)	—	35	1.9 (2.1)	—
EMA-captured hypoglycemic events	43	2.4 (2.4)	—	16	0.9 (1.0)	—

^a^TMB: TestMyBrain.org.

^b^RT: Reaction Time.

^c^Not available.

^d^DSM: Digit Symbol Matching.

^e^GradCPT: Gradual Onset Continuous Performance Test.

^f^MOT: Multiple Object Tracking.

^g^PSAT: Paced Serial Addition Test.

Paired samples 2-tailed *t* tests indicated that MOT accuracy was significantly better (*P*=.008) during the 3 EMA/day schedule than during the 6 EMA/day schedule (t_17_=2.96; *P*=.009; Cohen *d_z_*=0.70). For the other 5 cognitive tests, performance did not significantly differ between the 3 EMA/day and 6 EMA/day schedules (ChoiceRT medianRTc, *P*=.71; DSMmedianRTc, *P*=.57; FlickermedianRTc, *P*=.72; GradCPTD-Prime, *P*=.55; PSAT Accuracy, *P*=.75). The GradCPT reliability was much lower on the 6 EMA/day schedule than the 3 EMA/day schedule. This was because of the much higher between-person variance in scores (SD of scores) for the 3 EMA/day schedule than the 6 EMA/day schedule. However, the reason 3 EMA/day schedule captured more between-person variance in scores was unclear from our data.

EMAs were considered complete if the participants finished all 3 cognitive tests comprising the EMA. A paired samples 2-tailed *t* test found no significant difference in EMA completion rate between the 3 EMA/day schedule and the 6 EMA/day schedule (t_17_=1.16; *P*=.26; Cohen *d_z_*=0.27), with both schedules producing >80% EMA completion.

Another goal of the optimization pilot was to determine which EMA frequency schedule would be associated with more EMAs delivered within 60 minutes of a hypoglycemic event. Hypoglycemic episodes were considered only within 1 hour from the hours when EMAs were administered, that is, between 8 AM and 10 PM each day Hypoglycemia episodes were considered “captured” (overlapping with an EMA) if they met any of the following criteria: (1) any point of the hypoglycemic episode occurred within 60 minutes before the start of the EMA (2) the hypoglycemic episode began during the EMA, or (3) the hypoglycemic episodes began within 15 minutes following the end of the EMA. Of the 18 participants, 3 (17%) participants had no daytime hypoglycemic events during the entire pilot period (either 3 or 6 EMA/day periods, combined duration of wearing CGM for 15 d). During the 3 EMA/day period (10-day duration), 67% (12/18) of participants had 43 unique EMA-captured episodes of hypoglycemia (of 102 CGM-detected episodes). Two participants had no EMA-captured events despite having more than one event during the 10-day period. During the 6 EMA/day period (5-day duration), 50% (9/18) of participants experienced 16 unique EMA-captured episodes of hypoglycemia (out of 35 CGM-detected episodes). One participant had no EMA-captured events despite having >1 event during the 5-day period. When comparing periods within individual participants, 50% (9/18) of participants had more EMA-captured hypoglycemic events during the 3 EMA/day period compared to that of 6 EMA/day period, while only 11% (2/18) of participants had more EMA-captured events during the 6 EMA/day period. Overall, 39% (7/18) of participants had the same number of EMA-captured events in both periods.

A paired samples *t* test revealed that more hypoglycemic episodes occurred during the 3 EMA/day schedule than during the 6 EMA/day schedule (t_17_=3.00; *P*=.008; Cohen *d_z_*=0.71) as expected, given that the 3 EMA/day schedule spanned twice the amount of time as the 6 EMA/day schedule. Furthermore, more hypoglycemia episodes were captured by an EMA during the 3 EMA/day schedule (mean 2.4, SD 2.4) than during the 6 EMA/day schedule (mean 0.9, SD 1.0); t_17_=2.57; *P*=.02; Cohen *d_z_*=0.61 (Table 3).

## Discussion

### Implications for the Main GluCog Study

The optimization pilot was incorporated into the planned study design for the GluCog study to determine the EMA frequency that would (1) result in higher EMA completion and (2) capture more hypoglycemic events, which are critical factors in the success of the main study. On the basis of comparable EMA completion and greater EMA capture of hypoglycemic episodes, we selected 3 EMA/day schedule over the 6 EMA/day schedule for the main GluCog study.

The optimization pilot also allowed us to evaluate the psychometric and usability properties of the cognitive EMA measures. Given the paucity of cognitive EMA studies on which to base test selection, we initially selected 6 cognitive EMA measures within 3 cognitive domains and alternated tasks within each domain at each EMA (to reduce the total EMA duration). The data from the optimization pilot allowed us to select one test within each cognitive domain that had the greatest likelihood of producing usable data for the main GluCog study. For the processing speed domain, the Brief TMB DSM and Brief TMB Choice Reaction Time had comparable reliability and usability. We selected the Brief TMB DSM owing to greater familiarity among clinicians and the use of Digit Symbol Matching tasks in prior studies of cognition in T1D. For the sustained attention domain, we selected the Brief TMB GradCPT owing to less restriction of range when compared with the Brief Paced Serial Addition Test. For the working memory domain, we selected the Brief TMB MOT because some participants had technical issues with touch sensitivity on their devices during the Brief TMB Flicker task. Specifically, some participants had issues with screen taps not immediately registering on the device and as a result, had longer reaction times.

The optimization pilot also revealed technical difficulties in the implementation of the EMA via the app-based notification system. Some participants had trouble installing and setting up the app, as well as keeping track of push notifications. To address this, in the main GluCog study, we switched to a system that did not involve installation of an app and relied on text messages rather than push notifications. This system required less technical support throughout the study.

Many aspects of EMA study design can affect adherence to the research protocol [49,50]. The optimal assessment strategy for capturing sufficient glycemic variability and ensuring adequate EMA completion rates was unknown before initiating the GluCog study. The EMA cognitive tasks that we selected were also refined via an optimization pilot. We found no difference in the completion rates between the 2 EMA frequencies. However, we found that a longer sampling duration (10 d) with less frequent EMAs (3 EMAs per day) resulted in more EMAs in close proximity to hypoglycemic episodes compared with short duration of testing (5 days) with high-frequency EMAs (6 EMAs per day). Our findings may guide future studies that include events of interest with relatively infrequent occurrence (mean=0.51, SD =0.44 events per day; range 0-1.47), such as hypoglycemia.

### Other Considerations for the Main GluCog Study Procedure

Given that the onset of the COVID-19 pandemic coincided with recruitment for the optimization pilot, we were able to develop procedures for completing clinic visits remotely via telehealth or telephone visits, or in-person as initially planned, and carry these procedures forward into the main GluCog study, along with a COVID-19 specific impact and stress questionnaire. Recruitment was slower than initially anticipated owing to the rapidly increasing clinical uptake of real-time CGM in the T1D population. The exclusion of participants using real-time CGM was removed in June 2021 after 50 participants were enrolled to ensure that concurrent real-time CGM use could be analyzed as a covariate. The lower age limit was also expanded to 18 years to maximize recruitment. All the other procedures have remained consistent with those reported above. The main GluCog study began recruitment in October 2020. Enrollment was completed on June 15, 2022. Our primary objective was to describe the research methodology used in this pilot study on glycemic and cognitive variability in adults with T1D, the results of which guided key decisions related to EMA use in the main study protocol. There are limited existing cognitive EMA data on which important study-design decisions can be made. Recent systematic reviews on EMA have pointed out a lack of important methodological information in the scientific literature [6,49-51]. Our report includes all recommendations from the guidelines adapted for EMA studies across disciplines (Checklist for Reporting EMA Studies, CREMA) [6] and provides a way forward for EMA studies until greater methodological consensus has been reached.

### General Considerations for Other EMA Studies

The current availability of smartphones with large and high-resolution screens has made rigorous mobile cognitive assessments possible. Critical dilemmas faced by EMA studies involving cognitive assessment include (1) selecting tests that have been linked to the phenomena of interest based on the traditional test literature (ie, impacted cognitive domains); (2) ensuring psychometric properties that are suited to high-frequency administration (ie, avoiding ceiling effects); (3) balancing the need for adequate reliability, while minimizing test length; and (4) formatting a test that is compatible with smartphone screen size and operating system variations. There are very limited empirical data to aid the selection of EMA cognitive tasks that account for all these factors. Thus, there is an advantage in using a large web-based test platform (such as the TestMyBrain [21] platform) to select high-performing EMA length tests with known device impacts.

Although not anticipated when designing the GluCog study, our exclusive use of remote assessment was useful in mitigating logistical challenges related to face-to-face assessments during the COVID-19 pandemic [52,53]. We were able to quickly pivot our initial in-person clinic enrollment visit to a teleconference visit (with CGM supplies mailed to the participants). In addition, remote assessment mitigates logistical challenges related to traditional face-to-face testing administration, such as costs (staff time, clinic and laboratory space, physical assessment materials), difficulties in getting to a study site for people with mobility limitations or who have transportation challenges such as those who live in rural or remote areas, necessary training for the examiner, and training of study personnel in complex test administration (which can be particularly challenging with multisite studies).

Among the most exciting aspects of EMA use is the opportunity to explore biopsychosocial mechanisms underlying human health and disease. Technological advances in physiological detection capability (eg, CGM) and data-driven machine learning techniques for predicting cognitive changes are advancing rapidly. Our understanding of real-world influences on cognitive performance will be exponentially increased by the ability to accurately measure fluctuations as they occur. Repeated measurements allow for the identification of mediators and moderators of cognitive change over time in real-world environments.

### Conclusions

The EMA optimization pilot study described here responds to the urgent need for systematic and detailed information on EMA study designs. Recent advances in mobile technologies have resulted in new opportunities for EMA to examine cognition in everyday environments. When applied in a well-planned manner, EMA can be an important tool for research involving biopsychosocial mechanisms. In addition, cognitive EMA can assist in the early diagnosis of cognitive impairment as well as follow-up and intervention and can complement traditional neuropsychological assessments. EMA holds immense promise for understanding everyday conditions, both internal and external, that influence cognitive performance in individuals over time. This can be particularly useful in the assessment of populations with greater vulnerability to dynamic physiological, behavioral, and psychological interactions, such as individuals with T1D. Given the complexity of EMA studies, choosing the right instruments and assessment schedules is an important aspect of study design and subsequent data interpretation. Empirically determining these parameters in the target population will ensure adequate sampling of the phenomena of interest.

## Appendix

Multimedia Appendix 1 Ecological momentary assessment questions and tasks.

Multimedia Appendix 2 Score distribution across tests.

## Abbreviations

CGM: continuous glucose monitoring
DIA-LINK1: Towards a Better Understanding of Diabetes Distress, Depression and Poor Glycaemic Control
DSM: Digit Symbol Matching
EMA: ecological momentary assessment
FEEL-T1D: The Function and Emotion in Everyday Life with T1D
GluCog: Glycemic Variability and Fluctuations in Cognitive Status in Adults with Type 1 Diabetes
GradCPT: Gradual Onset Continuous Performance Test
Hypo-RESOLVE: Hypoglycaemia – Redefining Solutions for better lives
MOT: Multiple Object Tracking
T1D: type 1 diabetes
TMB: TestMyBrain.org
