# Supplementary material for: Glycemic Variability and Fluctuations in Cognitive Status in Adults With Type 1 Diabetes (GluCog): Observational Study Using Ecological Momentary Assessment of Cognition
Source: JMIR Diabetes. 2023 Jan 5;8:e39750. doi: 10.2196/39750 (PMC9853340; doi:10.2196/39750)
Supplement: Multimedia Appendix 1 [file diabetes_v8i1e39750_app1.docx]

|  | | | | | |
| --- | --- | --- | --- | --- | --- |
| EMA Patient Reported Questions | | | | | |
|  | Items | Questions | Based on (when applicable) | | Total time estimated |
| Recent Stress Exposure | 6 | Since the last survey:   - Did you have an argument or disagreement with someone? Yes / No - Did anything else happen that you could have argued or disagreed about, but you decided to let it pass? Yes / No - Did anything happen to a close friend or relative that turned out to be stressful for you? Yes/ No - Did anything stressful happen regarding your personal health? Yes / No - Did anything stressful happen regarding your diabetes? Yes / No - Did anything else happen that most people would consider stressful? Yes / No   [If yes to any of the above] How stressful was this experience? __ not at all stressful __ a little bit stressful __ somewhat stressful __ very stressful | Adapted from the Daily Inventory of Stressful Events.  Original: Almeida et al (2002)^46^  EMA format: Stawski et al (2008)^47^ | | 2 min |
| Anxiety and Depression | 7 | Since the last survey:   - How often have you felt: [EMOTION] __ none of the time __ a little of the time __ some of the time __ most of the time __ all of the time   Phrases will appear in place of [EMOTION] based on random selection / ordering of words from this list: Restless or fidgety, so sad that nothing could cheer you up, that everything was an effort, hopeless, irritated, worried, depressed | Adapted from: Sliwinski et al (2009)^48^ | |  |
| Alertness | )  1 | - Please indicate your sleepiness during the 5 minutes before this survey by choosing the appropriate description: 1=extremely alert 2=very alert 3=alert 4=rather alert 5=neither alert nor sleepy 6=some signs of sleepiness 7=sleepy, but no effort to keep awake 8=sleepy, some effort to keep awake 9=very sleepy, great effort to keep awake, fighting sleep | Adapted from: Akerstedt et al (2014)^49^ | |  |
| Contextual Factors | 3 | - There is a lot going on around me right now. __ Not at all true __ A little bit true __ Somewhat true __ Very true - I am having trouble concentrating right now. __ Not at all true __ A little bit true __ Somewhat true __ Very true - I am in a very noisy place right now. __ Not at all true __ A little bit true __ Somewhat true __ Very true | | |  |
| Glycemia | 4 | - Are you experiencing symptoms consistent with low blood glucose? Yes / No - Are you experiencing symptoms consistent with high blood glucose? Yes / No - What do you think your blood glucose is right now? __ very low __ low __ within range __ high __ very high - Why do you think that is your blood glucose? ___ symptoms ___ recent finger stick (last 30 minutes) ___ guessing | | |  |
| Alcohol and substance use | 1 | - Have you had alcohol or another substance that may affect your thinking since your last assessment? Yes/No.   [If yes]: What was the substance you ingested? __ alcohol __ marijuana ___ stimulant (other than caffeine) __ sleep medication ___ other (write-in)   - How long ago did you ingest alcohol or another psychoactive substance? __ hrs / min | | |  |
| Sleep | 1 | Morning EMA only:   - How well did you sleep last night? Very well / somewhat well / somewhat poorly / very poorly | | |  |
| EMA Cognitive Tasks | | | | | |
| Cognitive domain | | Content | Based on/ Validated by | Time | Total time estimated |
| Processing speed | | Brief TMB DSM  Brief TMB Choice RT | Hartshorne & Germine (2015)^36^  Singh et al. (2022)^50^ | 30 seconds | 3 minutes |
| Sustained Attention | | Brief TMB GradCPT  Brief TMB Flicker | Fortenbaugh, et al. (2015)^35^  Trevino et al. (2021)^51^ | 1 minute |  |
| Working memory | | Brief TMB MOT  Brief TMB PSAT | Trevino et al. (2021)^51^ | 1 minute |  |
