# Supplementary figures and images for: Glycemic Variability and Fluctuations in Cognitive Status in Adults With Type 1 Diabetes (GluCog): Observational Study Using Ecological Momentary Assessment of Cognition
Source: JMIR Diabetes. 2023 Jan 5;8:e39750. doi: 10.2196/39750 (PMC9853340; doi:10.2196/39750)

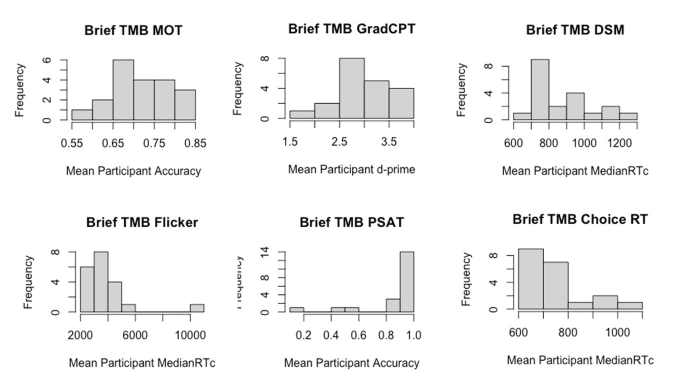

Supplement: Multimedia Appendix 2 [file diabetes_v8i1e39750_app2.png]
